# Supplementary material for: Gallionellaceae in rice root plaque: metabolic roles in iron oxidation, nutrient cycling, and plant interactions
Source: Appl Environ Microbiol. 2023 Nov 27;89(12):e00570-23. doi: 10.1128/aem.00570-23 (PMC10734482; doi:10.1128/aem.00570-23)
Supplement: Figures S1 to S4 — Supplemental Figures [file aem.00570-23-s0001.pdf]

Supplemental Materials

Gallionellaceae in rice root plaque: metabolic roles in iron oxidation, nutrient cycling and plant interactions

Clara S. Chan[1,2,3,4*], Gretchen E. Dykes[3,4,5^], Rene L. Hoover[1,3,4], Matt A. Limmer[5], Angelia L. Seyfferth[1,5*]

1 Dept. of Earth Sciences, University of Delaware, Newark, DE

2 School of Marine Science and Policy, University of Delaware, Newark, Delaware, USA

3 Microbiology Graduate Program, University of Delaware, Newark, DE

4 Delaware Biotechnology Institute, Newark, DE

5 Dept. of Plant and Soil Sciences, University of Delaware, Newark, DE

*corresponding authors cschan@udel.edu, angelias@udel.edu

^current address: U.S. Environmental Protection Agency, 1200 Pennsylvania Avenue NW, Washington, DC 20004

Number of pages: 6

Number of figures: 4

Number of tables: 5

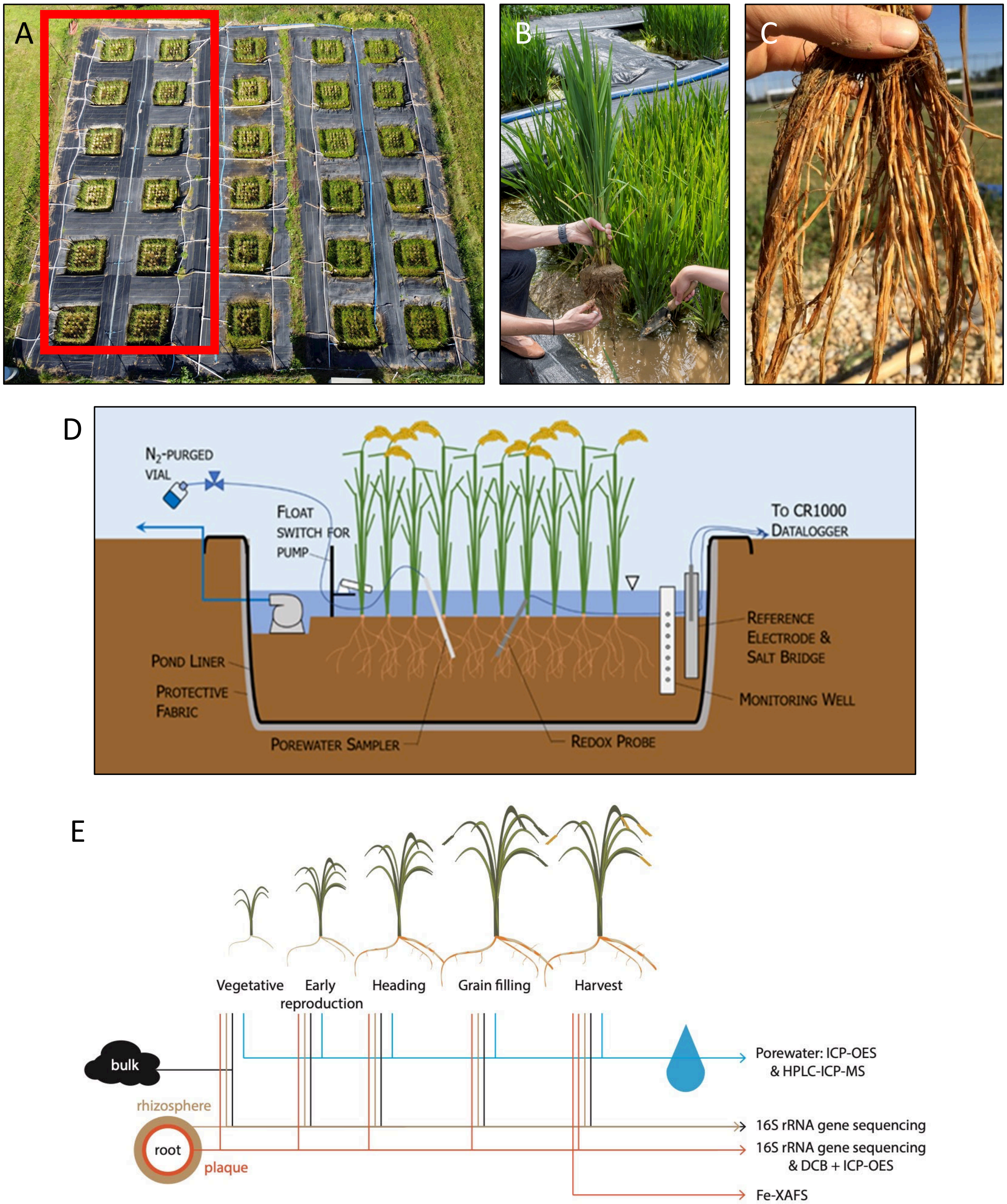

Figure S1. A) Overhead view of RICE facility, with the 12 paddies used in this study highlighted by the red rectangle. B) Individual rice plants were removed from each rice paddy throughout the growing season. C) Bulk soil and rhizosphere soil were removed from the plant to reveal the iron plaque. D) Schematic of an individual rice paddy. E) Timeline of plant and soil sampling, with specific analyses noted.

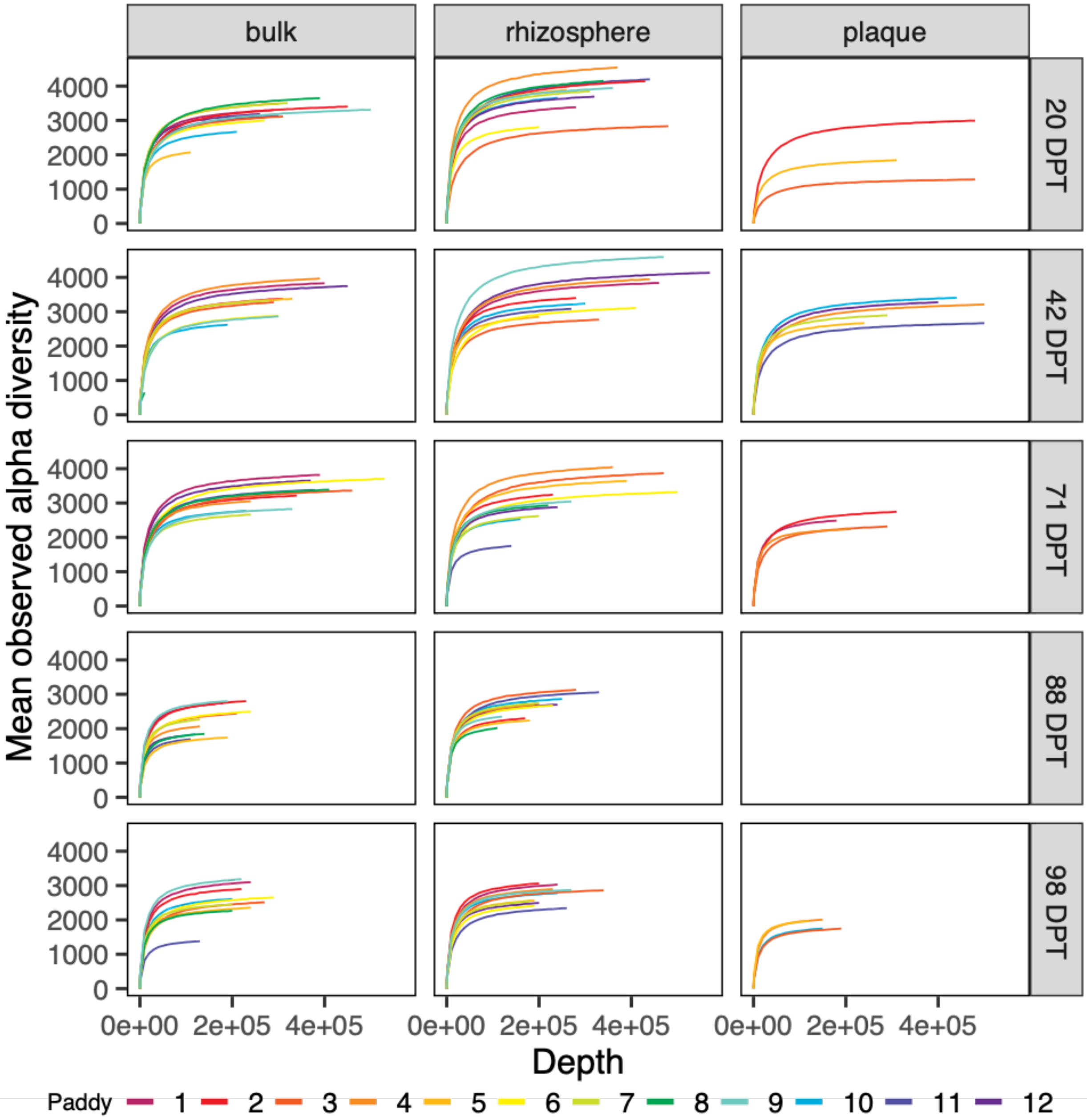

Figure S2. Observed alpha diversity rarefaction curves for each timepoint and biosphere. Rarefaction curves are colored by paddy.

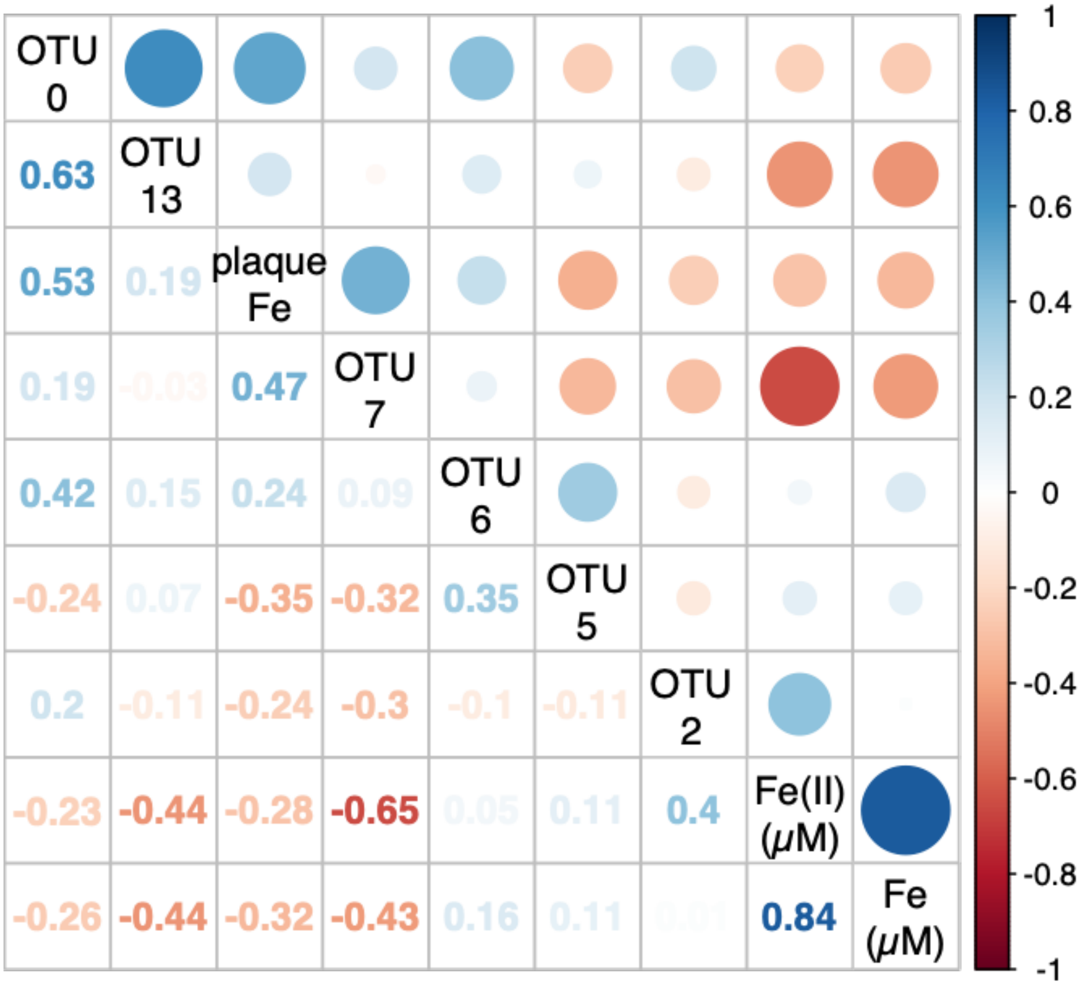

Figure S3. Spearman's rank correlation matrix of top Fe-cycling OTUs. Bubbles are sized and colored according strength and direction of Spearman correlation (see color scale), and rho is displayed on the lower left.

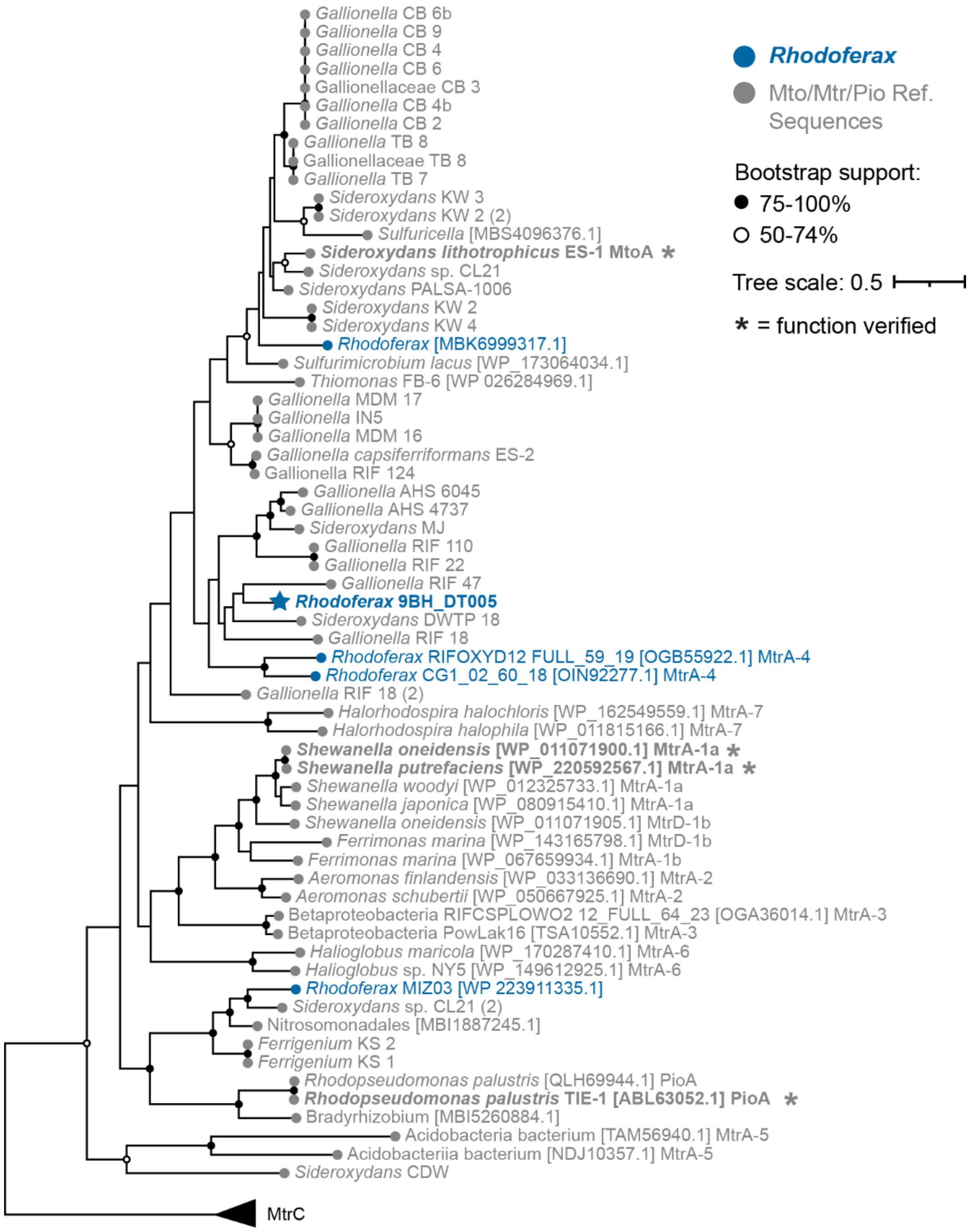

Figure S4. Maximum likelihood tree of MtoA/MtrA sequences showing that the *Rhodoferax* 9BH_DT005 sequence is closely related to functionally characterized iron oxidase MtoA sequences in *S. lithotrophicus* and within a clade of iron-oxidizing Gallionellaceae sequences. Support values based on 500 bootstraps. MtrA 1a, 1b, 2, 3, 4, 5, 6, and 7 indicate reference sequences from the seven MtrA groups defined by Baker, et al. (1).

Supplemental Tables

Available as a separate Excel file.

Table S1 OTU relative abundance and taxonomy

Table S2 Top plaque OTUs

Table S3 Genome average nucleotide and amino acid identity (ANI/AAI)

Table S4 CAZY genes

Table S5 Sampling time points for porewater and microbes

References

1. Baker IR, Conley BE, Gralnick JA, Girguis PR. 2022. Evidence for Horizontal and Vertical Transmission of Mtr-Mediated Extracellular Electron Transfer among the Bacteria. mBio https://doi.org/10.1128/mbio.02904-21.
